# Genetic Manipulation of Palmitoylethanolamide Production and Inactivation in *Saccharomyces cerevisiae*

**DOI:** 10.1371/journal.pone.0005942

**Published:** 2009-06-16

**Authors:** Giulio G. Muccioli, Angela Sia, Paul J. Muchowski, Nephi Stella

**Affiliations:** 1 Department of Pharmacology, University of Washington, Seattle, Washington, United States of America; 2 Louvain Drug Research Institute, Bioanalysis and Pharmacology of Bioactive Lipids Laboratory, Chemical and Physico-chemical Analysis of Drugs Unit, UCL-CHAM (7230), Université catholique de Louvain, Bruxelles, Belgium; 3 Gladstone Institute of Neurological Disease and Departments of Biochemistry and Biophysics, and Neurology, University of California San Francisco, San Francisco, California, United States of America; 4 Department of Psychiatry and Behavioral Sciences, University of Washington, Seattle, Washington, United States of America; Auburn University, United States of America

## Abstract

**Background:**

Lipids can act as signaling molecules, activating intracellular and membrane-associated receptors to regulate physiological functions. To understand how a newly discovered signaling lipid functions, it is necessary to identify and characterize the enzymes involved in their production and inactivation. The signaling lipid *N*-palmitoylethanolamine (PEA) is known to activate intracellular and membrane-associated receptors and regulate physiological functions, but little is known about the enzymes involved in its production and inactivation.

**Principal Findings:**

Here we show that *Saccharomyces cerevisiae* produce and inactivate PEA, suggesting that genetic manipulations of this lower eukaryote may be used to identify the enzymes involved in PEA metabolism. Accordingly, using single gene deletion mutants, we identified yeast genes that control PEA metabolism, including *SPO14* (a yeast homologue of the mammalian phospholipase D) that controls PEA production and *YJU3* (a yeast homologue of the mammalian monoacylglycerol lipase) that controls PEA inactivation. We also found that PEA metabolism is affected by heterologous expression of two mammalian proteins involved in neurodegenerative diseases, namely huntingtin and α-synuclein.

**Significance:**

Together these findings show that forward and reverse genetics in *S. cerevisiae* can be used to identify proteins involved in PEA production and inactivation, and suggest that mutated proteins causing neurodegenerative diseases might affect the metabolism of this important signaling lipid.

## Introduction

Lipids are the building blocks of cell membranes, and their turnover is regulated by specific enzymes, including lipases and acyltransferases. Stimuli that regulate the activity of these enzymes may transiently change lipid turnover and cause the release of specific lipids from cell membranes. Released lipids may then activate intracellular and membrane-associated receptors, and thus act as second messengers, transmitters and hormones. The biological actions of released lipids are terminated by hydrolysis or chemical modification. Thus, since the biological action of released lipids depends on their rate of production and inactivation, the enzymatic steps involved in these processes must be fully appreciated to understand their signaling function.

The endocannabinoids (eCBs) *N*-arachidonoylethanolamine (anandamide) and 2-arachidonoylglycerol (2-AG) are signaling lipids that act through membrane-associated cannabinoid receptors and regulate neurotransmission, inflammation, and other physiopathological functions [1]. Anandamide is an *N*-acylethanolamine (*N*-AE) and 2-AG is a glycerolacylester. Both contain a C20:4 acyl chain, but their respective polar groups impinge distinct pharmacological profiles at cannabinoid receptors [2]. PEA, a signaling lipid of the *N*-AE family, contains a C16:0 acyl chain and acts on its own membrane-associated and intracellular receptors, including Gpr55 and PPARα [3], [4]. Thus, while anandamide, 2-AG, and PEA are structurally related, these lipids possess different bioactivities, and unraveling their respective functions will require a better understanding of the molecular mechanisms controlling their production and inactivation.

Anandamide, 2-AG, and PEA may be independently released from cell membranes [5], [6], suggesting that independent enzymes and molecular mechanisms control their production. To date, at least four enzymes have been implicated in anandamide production: a subtype of phospholipase D (Nape-Pld), phospholipase C (Plc), phosphatase (Ptpn22) and hydrolase (α/β-hydrolase 4) [7]–[9]. Four enzymes have been implicated in 2-AG production: Plc-β, α- and β- diacylglycerol lipase, and lyso-Plc [10]–[13]. Only one enzyme has been implicated in PEA production: Nape-Pld [14]. Thus, little is known about PEA production.

Anandamide is predominantly inactivated by fatty acid amide hydrolase (Faah) and, under certain conditions, by cyclooxygenase, lipooxygenase, and *N*-acylethanolamine-hydrolyzing acid amidase (Naaa) [15]–[19]. 2-AG is inactivated by monoacylglycerol lipase (Mgl), triacylglycerol lipase, cyclooxygenases, lipooxygenases, and the newly identified α/β-hydrolase 6, α/β-hydrolase 12 (Abhd12), and Neuropathy target esterase [20]–[25]. PEA is inactivated by Faah and Naaa [16], [26]–[27] and possibly by a unidentified enzyme expressed by microglial cells [28]. Thus, at least one enzyme that inactivates PEA remains to be identified at the molecular level.

*Saccharomyces cerevisiae* has been extensively used as genetic tool to dissect metabolic pathways, including those involved in lipid production and inactivation. Although these cells have no genes encoding for receptors activated by anandamide and 2-AG, they express Oaf1/Pip2, a functional homologue of PPARα activated by PEA [29]. Furthermore, *S. cerevisiae* expresses enzymes closely related to those which produce and inactivate *N*-AEs and glycerolacylesters in mammalian cells (Table 1). Thus, it is possible that these lower eukaryotes may also produce and inactivate anandamide, 2-AG and PEA. If so, genetic screens could be used to systematically identify genes controlling their production and inactivation.

**Table 1:** Humans orthologs of yeast genes coding for selected proteins involved in lipid metabolism in yeast.

Yeast gene	Putative function in yeast	Human gene	Protein sequence identity
*LRO1*	Acyltransferase	LyPla3	31%
*PLB1*	Phospholipase B	Pla2	24%
*PLB2*	Phospholipase B	Pla2	26%
*PLB3*	Phospholipase B	Pla2	24%
*PLC1*	Phospholipase C	Plcd4	31%
*ISC1*	Phospholipase C	Smpd2	28%
*SPO14*	Phospholipase D	Pld1	44%
*YJU3*	Serine hydrolase activity	Mgl	24%
*AMD2*	Amidase	Faah	27%
*FSH1*	Serine hydrolase	Ovca2	27%

The table shows example of yeasts gene coding for proteins involved in lipid metabolism. Their human hortolog are listed with the sequence identity between the yeast and the human protein.

Here we addressed these possibilities by determining if *S. cerevisiae* produce anandamide, 2-AG and PEA (as well as several additional related *N*-AEs). We also tested whether single gene deletions or heterologous expression of mammalian proteins affects the production and inactivation of such signaling lipids.

## Results

We used chemical ionization gas chromatography mass spectrometry (CI-GC-MS) to determine if yeast produce anandamide, 2-AG, PEA, as well as several other bioactive *N*-AEs: stearoylethanolamide (SEA), oleoylethanolamide (OEA), homo-γ-linolenoylethanolamide (HEA) and docosatetraenoylethanolamide (DEA). SEA acts through an unknown receptor, OEA acts through PPARα, while HEA and DEA are closely related to anandamide and also act on CB_1_ receptors [30]–[34].

### Yeast Produce PEA

**Figure 1:**
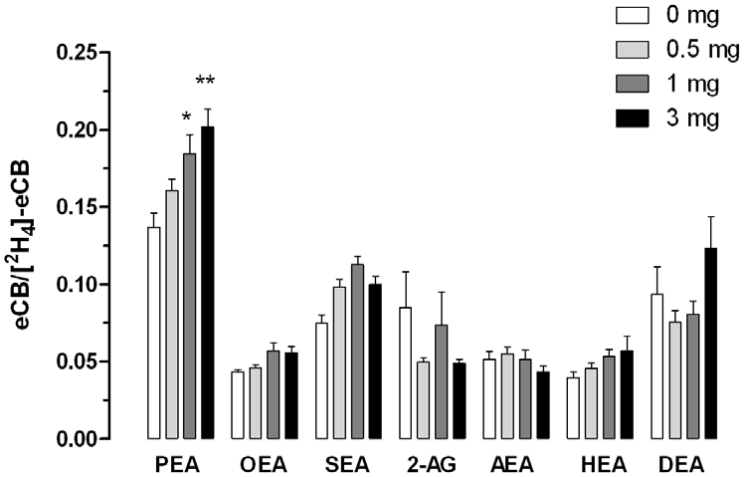
Yeast produces PEA. Increasing amounts of yeast homogenate (0, 0.5, 1, and 3 mg of protein *per* sample) were analyzed by CI-GC-MS and the non-deuterated/deuterated ratios determined using 200 pmol of [^2^H_5_]-2-AG and [^2^H_4_]-*N*-AEs standards. Values are the mean±SEM of three to four independent experiments, performed in duplicate, and demonstrate that only PEA is reliably detected in yeast homogenates using this method. * P<0.05 and ** P<0.01 compared to the ratios obtained in the absence of yeast homogenate (ANOVA one-way, Dunnett's post test).

To identify and quantify the levels of these lipids, we spiked yeast homogenates with corresponding deuterated standards and performed isotope-dilution quantifications [35]. Since deuterated standards contain trace amounts of the lipid itself (typically a few percent), we first determined the amount of yeast homogenate required to generate an ion current ratio that is statistically greater than that generated by the standards alone. Yeast cells were grown to saturation and homogenized, and the total protein concentrations quantified. Total homogenate (0.5, 1, and 3 mg of total protein) was chloroform-extracted in the presence of 200 pmol of the deuterated standards and analyzed by isotope-dilution CI-GC-MS. We found that the ion current ratios of PEA/[^2^H_4_]-PEA generated by 1 and 3 mg of yeast homogenates were the only ones statistically greater than those generated by standards alone (Figure 1). Denser samples led to chromatography overload, precluding quantitative analysis. Conversion of the ratios showed that yeast produce 0.65 pmol of PEA *per* mg of protein, well within the range generated in mammalian tissue [35].

### The Lipase Spo4 Contributes to PEA Production

**Figure 2:**
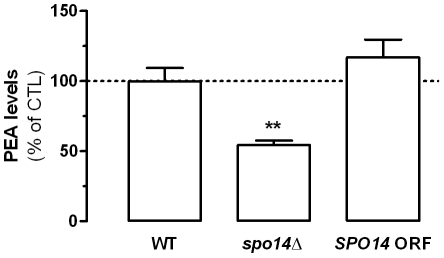
Spo14 is involved in PEA production. PEA levels in *SPO14* gene-deleted yeast strain (*spo14*Δ) are compared to wild-type strain (WT), and to the PEA levels measured in a *SPO14* gene-deleted yeast strain in which an *SPO14* ORF was reintroduced (*SPO14* ORF). Values are the mean±SEM of three experiments, performed in duplicate. ** P<0.01 compared to PEA levels obtained for WT yeast (ANOVA one-way, Dunnett's post test).

In the next series of experiments, we used a candidate gene approach and selected ten yeast strains with single gene deletions for various lipases and acyltransferases (Table 1), and determined the effects of these mutations on PEA production compared to wild type yeast. Figure 2 shows that PEA production was reduced by 46% in *spo14*Δ cells, which lack a phospholipase D-like activity, and that this reduction was fully rescued when this gene was reintroduced. None of the other mutations affected PEA levels (Figure S1). These results suggest that the phospholipase D-like activity of Spo14 is responsible for producing PEA in yeast.

### Yeast Hydrolyze PEA

**Figure 3:**
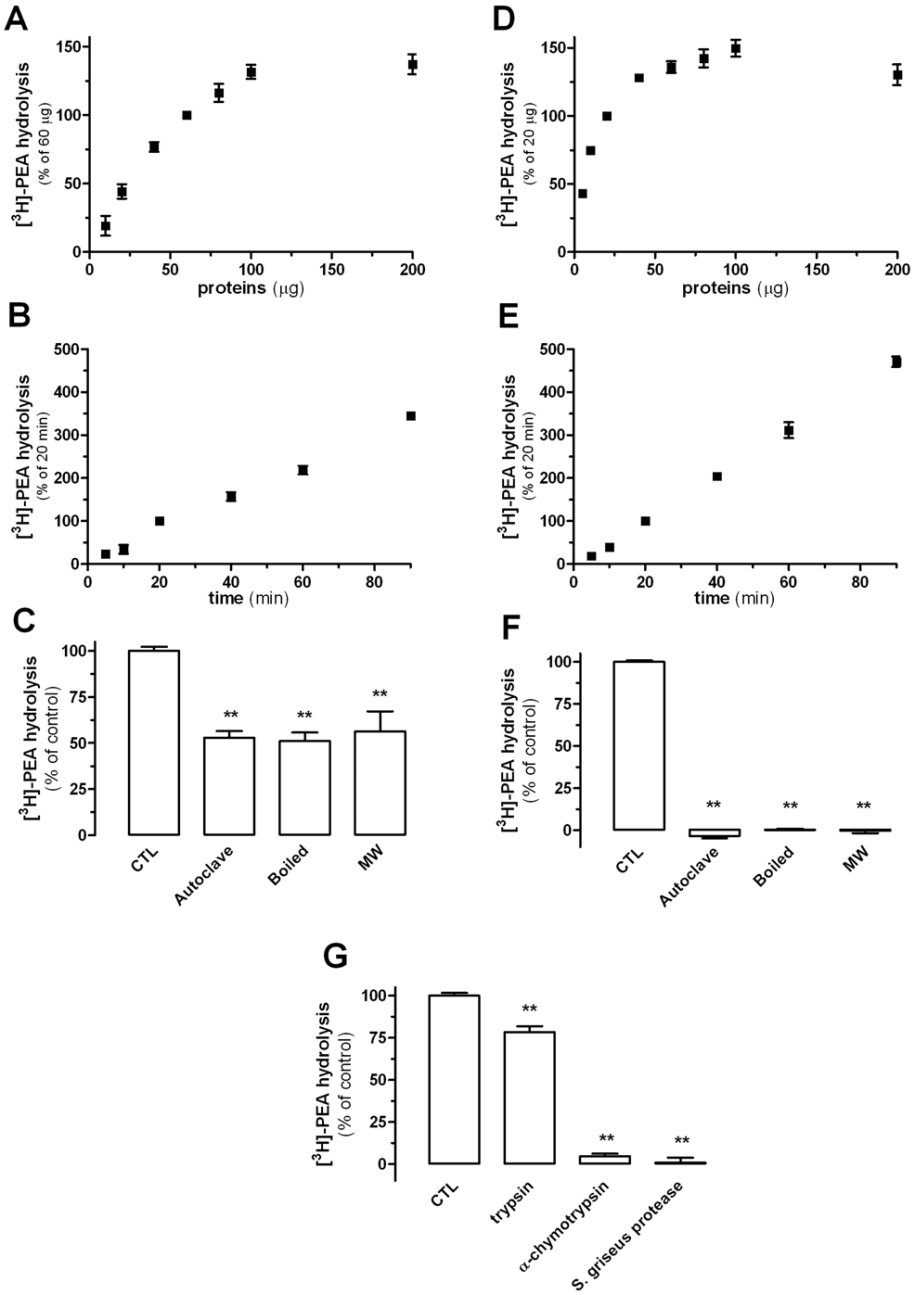
Initial characterization of PEA Hydrolysis by Yeast Homogenates. [^3^H]-PEA hydrolysis by yeast homogenates is protein concentration-dependent (A; 20 min incubation) and time-dependent (B; 60 µg of protein). Similarly, HEK293 cells homogenates hydrolyze [^3^H]-PEA in a protein concentration- (D; 20 min incubation) and time-dependent manner (E; 20 µg of protein). When submitted to denaturing conditions such as heat or microwaves, ≈50% of the yeast PEA-hydrolytic activity (C) and ≈100% of the HEK293 cells homogenate activity (F) are lost, suggesting that part of the hydrolytic activity in yeast in heat-resistant. [^3^H]-PEA hydrolysis is greatly reduced in yeast homogenates incubated with α-chymotrypsin or with protease from *Streptomyces griseus* (G) confirming the enzymatic nature of the entire PEA hydrolysis. Data points are the mean±SEM of three to four experiments done in duplicate. ** P<0.01 compared to control homogenates (ANOVA one-way, Dunnett's post test).

Next we assessed the ability of yeast to inactivate PEA. Indeed, yeast homogenates hydrolyzed [^3^H]-PEA in a concentration- and time-dependent manner, yielding a specific activity of 5.0×10^−3^ pmol/min/mg of protein that reached saturation when using 100 µg of protein (Figure 3A and 3B). To ascertain that a protein was responsible for PEA hydrolysis, we subjected yeast homogenates to heat-induced denaturation. Boiling or autoclaving these samples reduced hydrolysis by only ∼50% (Figure 3C). Since some enzymes produced by lower organisms are highly resistant to heat-induced denaturation [36], we also microwaved the homogenates. This regimen also reduced [^3^H]-PEA hydrolysis by ∼50% (Figure 3C). As positive control for these experiments, we used homogenates of human embryonic kidney cells HEK293 cells. These cells hydrolyzed [^3^H]-PEA in a protein-concentration and time-dependent manner, yielding a specific activity of 3.9×10^−2^ pmol/min/mg of protein that reached saturation when using 40 µg of protein (Figure 3D,E). This activity was lost when homogenates were boiled, autoclaved, or microwaved (Figure 3F).

To determine whether both the heat-sensitive and heat-resistant PEA hydrolysis in yeast were due to proteins, we treated the yeast homogenates with trypsin, α-chemotrypsin, and a protease from *Streptomyces griseus*. Figure 3G shows that both α-chemotrypsin and the *Streptomyces griseus* protease reduced the entire PEA hydrolysis activity by 96% and 99%, respectively, showing that both heat-sensitive and heat-resistant activities are due to proteins.

### A Serine Hydrolase Distinct From FAAH Hydrolyzes PEA

**Figure 4:**
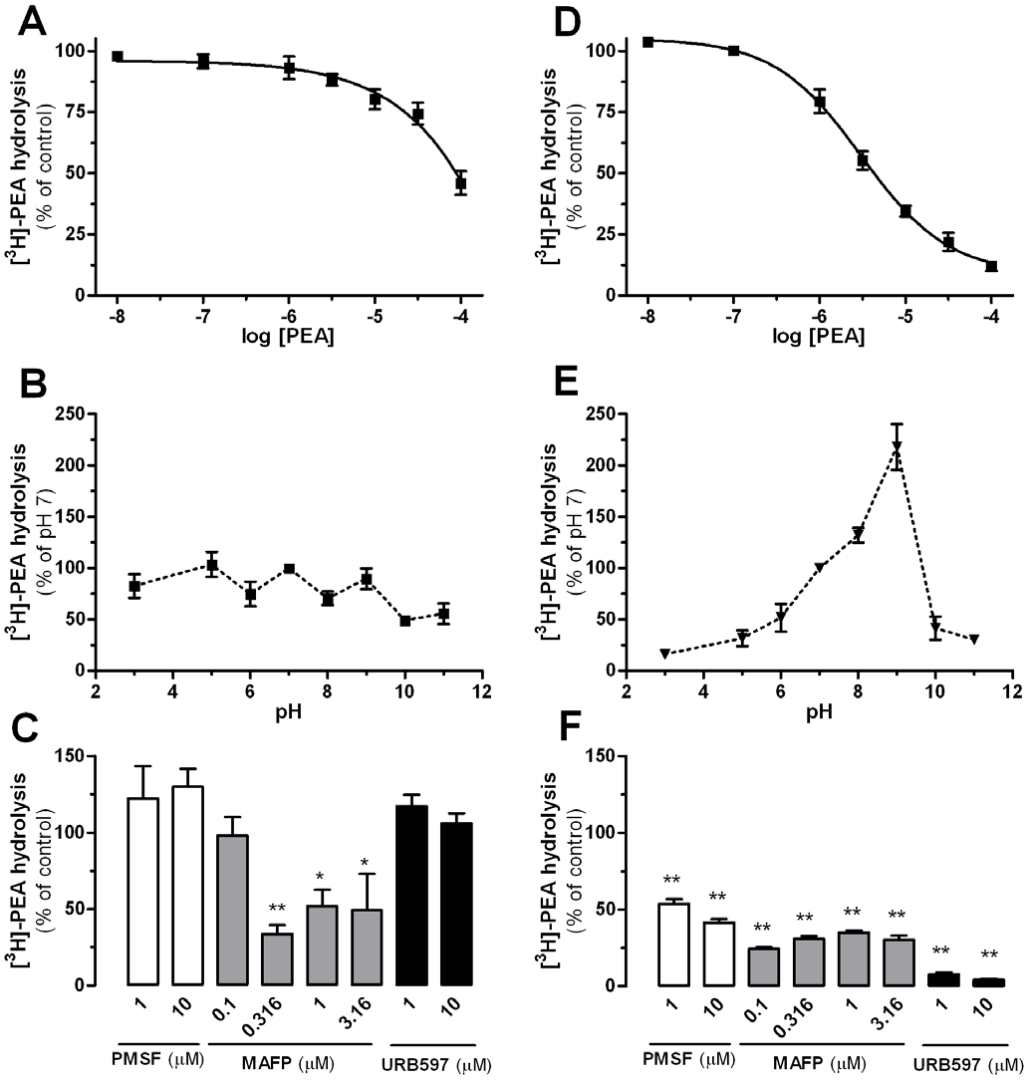
Further characterization of PEA Hydrolysis by Yeast Homogenates. The effect of increasing concentrations of PEA on [^3^H]-PEA hydrolysis by yeast (A) and HEK293 cells (D) homogenates and the influence of the pH on [^3^H]-PEA hydrolysis by yeast (B) and HEK293 cells (E) homogenates were determined. The following buffers were used (100 mM): NaAcetate (pH 3–5), HEPES (pH 6–7), Tris (pH 8–9) and Na_2_B_4_O_7_ (pH 10–11). The serine hydrolase inhibitors PMSF and MAFP and the specific Faah inhibitor URB597 differentially affected [^3^H]-PEA hydrolysis by yeast (C) and HEK cells (F) homogenates. Data points represent the mean±SEM of 3 to 5 experiments performed in duplicate and are expressed as percentage of control. * P<0.05 and ** P<0.01 compared to control homogenates (ANOVA one-way, Dunnett's post test).

We performed an initial biochemical characterization of the enzymatic activity responsible for [^3^H]-PEA hydrolysis. In yeast, unlabelled PEA inhibited this activity in dose-dependent manner, reaching ∼50% inhibition at 100 µM PEA (Figure 4A). In HEK293 homogenates, unlabelled PEA competed for [^3^H]-PEA hydrolysis, reaching 50% inhibition at 3.1 µM and 90% inhibition at 100 µM PEA (Figure 4D). [^3^H]-PEA hydrolysis by yeast homogenates was marginally affected by changes in pH (Figure 4B), suggesting that several yeast enzymes can hydrolyze PEA, each at their optimal pH value. In contrast, hydrolysis by HEK293 cells was clearly pH dependent, increasing by 10-fold between pH 3 and 9 (Figure 4E), suggesting hydrolysis by a single enzyme with optimal activity at pH 9. One such enzyme is Faah [26], [27], [37].

Three chemically distinct inhibitors (PMSF, MAFP, and URB597) were tested on PEA hydrolysis by both yeast and HEK293 cell homogenates. While both PMSF and URB597 were inactive on [^3^H]-PEA hydrolysis by yeast homogenates, MAFP at 300 nM inhibited 50% of this activity, suggesting the involvement of a serine hydrolase (Figure 4C). In HEK293 cells homogenates, PMSF and MAFP partially inhibited [^3^H]-PEA hydrolysis, leaving approximately 30% of the activity intact, while URB597 fully inhibited [^3^H]-PEA hydrolysis (Figure 4F). These results suggest that the enzyme(s) responsible for PEA hydrolysis in yeast is/are most likely a serine hydrolase(s) distinct from Faah, whereas Faah is likely the main enzyme responsible for PEA hydrolysis in HEK293 cells.

### The Serine Hydrolase Yju3 Contributes to PEA Hydrolysis

**Figure 5:**
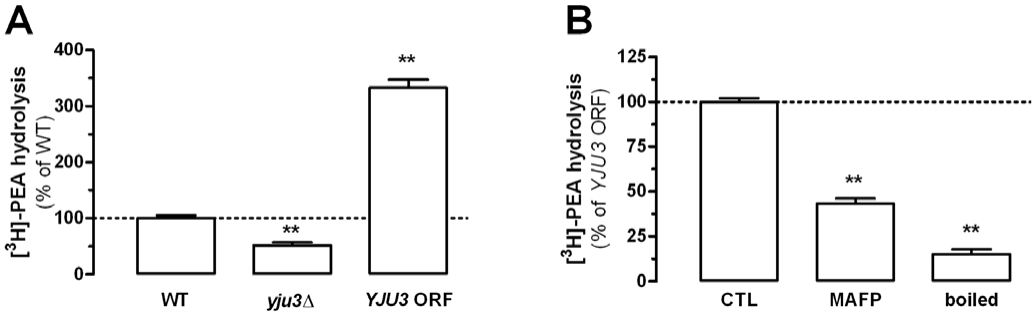
Yju3 is involved in PEA hydrolysis. [^3^H]-PEA hydrolysis in *YJU3* gene-deleted yeast strain (*yju3*Δ) is compared to wild-type strain (WT), and to the hydrolysis measured in a *YJU3* gene-deleted yeast strain in which an *YJU3* ORF was reintroduced (*YJU3* ORF) (A). The [^3^H]-PEA hydrolysis found in the Yju3 expressing strain (i.e. *YJU3* ORF expressing strain (CTL)) is partially sensitive to MAFP inhibition and sensitive to denaturing conditions (boiled) (B). Values are the mean±SEM of three experiments performed in duplicate and are expressed as percentage of control. ** P<0.01 compared to control (ANOVA one-way, Dunnett's post test).

When testing the ten yeast strains with single gene mutations that we had selected (Table 1), we found that PEA hydrolysis was reduced by 49% in *yju3*Δ, a strain that lacks a serine hydrolase orthologous to the mammalian MGL (Figure 5A). This reduction in activity was not only rescued when the *YJU3* gene was reintroduced, but PEA hydrolysis was actually greatly increased (Figure 5A). This increased PEA hydrolyzing activity exhibited by Yju3-bearing yeast strains was reduced by 57% by MAFP and by 85% when its homogenates were heat-inactivated (Figure 5B), suggesting that the MGL-like activity of Yju3 is responsible for the heat-sensitive PEA hydrolyzing activity expressed by yeast.

Interestingly, *plc1*Δ and *isc1*Δ, two strains deficient in Plc-like activity, led to a small, but significant increase in PEA hydrolysis, suggesting compensatory mechanisms (Figure S1). Furthermore, genetic deletion of *AMD2*, a yeast homologue of Faah, did not affect PEA production or inactivation (Figure S1), which strengthens the conclusion that PEA is hydrolyzed by a yeast enzyme without Faah-like activity.

### Mutant Huntingtin and α-Synuclein Affect PEA Production and Inactivation

Because several mammalian proteins involved in the pathogenesis of neurodegenerative diseases are known to affect lipid metabolism [38], and several neuropathologies lead to changes in PEA levels in brain [39], we expressed mutant huntingtin (Htt) fragments and α-synuclein in yeast and assessed their effect on PEA production and inactivation.

**Figure 6:**
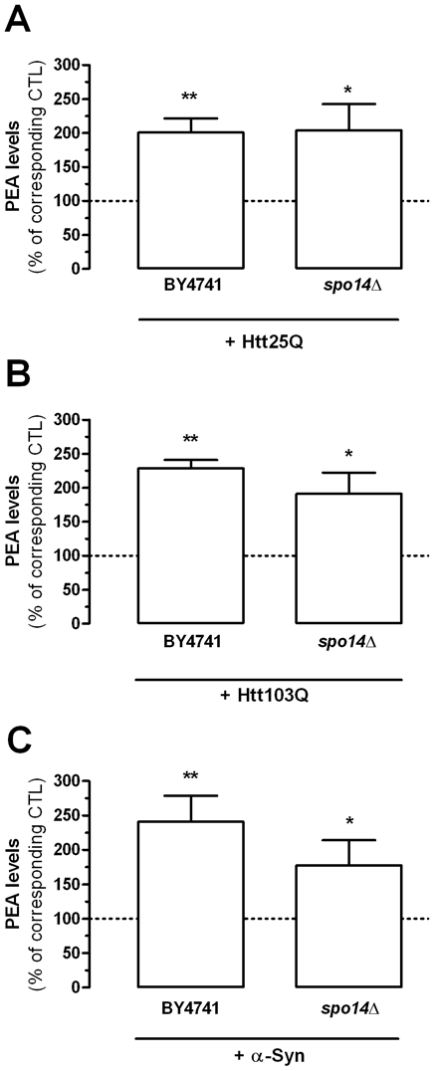
Htt fragments and α-synuclein increase PEA production in yeast. PEA levels found in BY4741 yeast strains expressing a 25Q and 103Q huntingtin fragment and in yeast strain expressing α-synuclein (BY4741) were compared to BY4741 strains expressing an empty vector (dotted line). Similarly PEA levels found in *SPO14* gene-deleted yeast strains expressing the same proteins (*spo14Δ*) were compared to *spo14Δ* strains expressing an empty vector (dotted line) (A–C). Values are the mean±SEM of three to five experiments performed in duplicate and are expressed as percentage of corresponding control (empty vector expressing BY4741 strain and empty vector expressing *spo14Δ* strain, respectively). ** P<0.05 and ** P<0.01 compared to respective control (unpaired two-tailed Student's t-test).

Htt fragments containing 25 and 103 polyglutamine repeats (25Q and 103Q), as well as α-synuclein, each increased by ∼2 fold the levels of PEA (Figure 6A–C), whereas the empty vector had no significant effect (data not shown). To determine if this increase in PEA production involves Spo14, we expressed Htt fragments and α-synuclein in the *spo14*Δ strain, and measured PEA levels. Remarkably, PEA levels were also increased by ∼2 fold by these pathogenic fragments and proteins in this strain (Figure 6A–C), suggesting that they induce the expression of another PEA producing enzyme.

**Figure 7:**
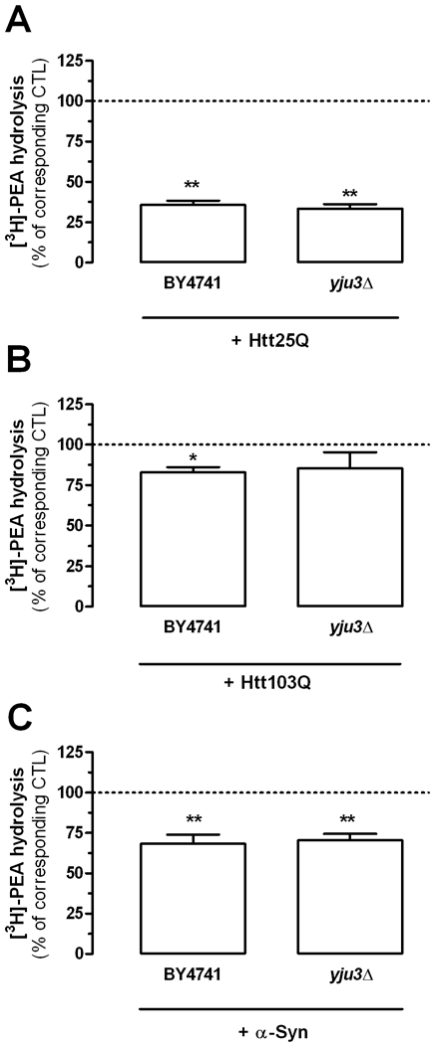
Htt fragments and α-synuclein reduce PEA hydrolysis in yeast. [^3^H]-PEA hydrolysis measured in BY4741 yeast strains expressing a 25Q and 103Q huntingtin fragment and in yeast strain expressing α-synuclein (BY4741) were compared to BY4741 strains expressing an empty vector (dotted line). Similarly [^3^H]-PEA hydrolysis found in YJU3 gene-deleted yeast strains expressing the same proteins (*yju3Δ*) were compared to *yju3Δ* strains expressing an empty vector (dotted line) (A–C). Values are the mean±SEM of three to five experiments performed in duplicate and are expressed as percentage of corresponding control (empty vector expressing BY4741 strain and empty vector expressing *yju3Δ* strain, respectively). ** P<0.05 and ** P<0.01 compared to respective control (unpaired two-tailed Student's t-test).

With regard to PEA hydrolysis, Htt25Q reduced it by 65%, Htt103Q had a small (17%), but significant effect, and α-synuclein reduced this activity by 32% (Figure 7A–C). The empty vector had no significant effect (data not shown). To determine if this decrease in PEA hydrolysis involves Yju3, we expressed Htt fragments and α-synuclein in the *yju3*Δ strain. Here too, PEA hydrolysis was similarly decreased by these pathogenic fragments and proteins (Figure 7A–C), suggesting that they reduce the expression of another PEA hydrolyzing enzyme.

## Discussion

PEA is a highly bioactive lipid that activates intracellular and membrane-bound receptors distinct from those activated by eCBs. The molecular machinery controlling PEA production and inactivation in mammalian cells is not fully understood [40]–[43]. Our results show that *S. cerevisiae* produce and inactivate PEA, and that this process is significantly altered by single gene deletions or heterologous expression of mammalian proteins involved in neurodegenerative diseases. Thus, our findings show that forward and reverse genetics in yeast can be used to identify the molecular components that control PEA production and inactivation.

Using CI-GC-MS and isotope-dilution, we demonstrated that *S. cerevisiae* produce significant amounts of PEA, a lipid that contains a C16:0 acyl chain, while not producing detectable amounts of anandamide, 2-AG, OEA, SEA, HEA, and DEA, all of which contain acyl chains with more than 16 carbon atoms. Accordingly, classic studies showed that lower eukaryotes cannot produce fatty-acid chains containing more than 16 carbon atoms despite the presence of two enzymes, fatty acid elongase and enoyl reductase, that are known to lengthen carbon acyl chains [44]–[46].

The amount of PEA we detected in yeast (650 pmol/g) is comparable to that in mouse brain (100–500 pmol/g) and cultured neural cells (1800–2200 pmol/g of protein) (for review see [35]). However, reliable PEA quantification required only 3 mg of yeast homogenate sample but 30 mg of mouse brain homogenate [35]. This 10-fold difference suggests that the polysaccharide wall in yeast is less invasive when analyzing PEA by CI-GC-MS than the rich lipidic environment of the brain.

PEA is also produced and hydrolyzed by plants, where it is thought to be a response mechanism to stressors and regulator of plant growth [47]. PEA was also quantified in *Ciona intestinalis* and in leech [48]. Together with our results, these studies show that PEA is produced by a wide variety of cell types and organisms. Whether PEA constitutes a building block of yeast membranes or carries the function of signaling lipid in these lower eukaryotes remains to be determined. Note that while we do not suggest that the function of PEA as a signaling lipid in mammalian cells is conserved in yeast, our data indicate that the basic machinery involved in its production and inactivation might be conserved, and therefore yeast constitute an incredibly efficient genetic tool to dissect the molecular mechanisms involved in PEA metabolism.

Genetic evidence demonstrating the involvement of specific enzymes in PEA production and inactivation is sparse. To our knowledge, only two examples have been reported: Nape-Pld, which affects PEA production, and Faah, which affects PEA inactivation [14], [26]. By screening 10 yeast gene deletion strains, we identified two additional candidates: Spo14 (coding for a Pld-like activity) and Yju3 (coding for a Mgl-like serine hydrolase activity). These genes have mammalian orthologs: Spo14 is related to Pld1 (coding for a phosphatidylcholine-hydrolyzing Pld1), and Yju3 is related to Mgl (coding for a monoglyceride lipase) and to some extent to Abhd12 (coding for a serine hydrolase recently implicated in eCB signaling [25]). The implication of a yeast homologue of Mgl and Abhd12 in PEA hydrolysis is remarkable for two reasons. First, it is known that mammalian Mgl does not hydrolyze PEA [21], suggesting that the mammalian Mgl evolved so as to preferentially hydrolyze acylesters rather than acylamide. Second, the substrate specificity of Abhd12 is still unknown, and our data suggest that this newly discovered serine hydrolase might use PEA as substrate and thus be involved in its inactivation in mammalian cells.

Approximately 50% of the PEA hydrolysis expressed by yeast is resistant to heat and microwave inactivation, but is fully blocked by enzymatic digestion. This result agrees with the fact that enzymes resistant to heat-induced denaturation are expressed by some lower organisms [36]. While the identity of this heat-resistant enzyme expressed by yeast remains to be determined, our results already suggest that it is unlikely to be a serine hydrolyze, since it is insensitive to the broad serine-hydrolase inhibitor MAFP.

We found that heterologous expression of huntingtin fragments 25Q and 103Q in yeast increased PEA level by ∼2 fold, showing that the ability of huntingtin to control PEA levels does not depend on the length of the polyglutamine repeat that determines the age of onset of Huntington's disease. Considering the proposed role of huntingtin in the regulation of gene expression [49], this result suggests that increased PEA levels measured here might not be due to a direct involvement of huntingtin in PEA metabolism, but rather due to huntingtin-mediated impairment of transcription of key molecular steps controlling PEA levels (*e.g.* increased expression of enzymes producing PEA or decreased expression of enzymes inactivating PEA) [50]. We also found that α-synuclein expression increased PEA levels by ∼2 fold, suggesting that the metabolism of this signaling lipid might also be impaired in patients with Parkinson's disease carrying this mutation, as well as mice models carrying this mutation. To our knowledge, no such measurement has been reported, even though α-synuclein is known to affect lipid metabolism [51], [38].

The molecular steps occurring between the presence of huntingtin and α-synuclein in yeast, and a change in PEA metabolism remain to be determined, since performing these experiments in yeast strains lacking Spo14 and Yju3 did not prevent the effects of these pathogenic proteins, suggesting that they regulate the expression of other proteins. Here, two points can be made. First, it is unlikely that the increase in PEA levels induced by Htt25Q, Htt103Q and α-synuclein results from the down-regulation of a PEA inactivating enzymes, since we found that the genetic mutation of *YJU3* leads to a 49% reduction in PEA hydrolysis without increasing PEA levels (compare Fig 5a and Figure S1). Second, the molecular mechanism involved in these changes in PEA metabolism could be dissected out by using combinations of the forward and reverse yeast genetic approaches described here.

In summary, identifying the molecular steps that control the production and inactivation of signaling lipids is essential for understanding their biological functions and for developing new therapeutic approaches. We found that *S. cerevisiae* produce and inactivate PEA, and identified several yeast genes involved in its production and inactivation. PEA metabolism in *S. cerevisiae* was affected by heterologous expression of mammalian proteins involved in Huntington's disease and Parkinson's disease, suggesting that PEA signaling is affected in those conditions. Thus, our results provide proof-of-concept for the use of reverse and forward yeast genetics to gain a genome-wide understanding of the molecular steps in PEA metabolism.

## Materials and Methods

### Materials

All solvents were of analytical grade. URB597, MAFP, 2-AG, and [^2^H_5_]-2-AG were from Cayman Chemical (Ann Arbor, MI), PMSF, trypsin, chemotrypsin, and Streptomyces griseus proteases were from Sigma (St Louis, MO). [^3^H]-PEA (radioactively labeled on the ethanolamine) was from American Radiolabeled Chemicals (St. Louis, MO) and the NIDA drug supply system. *N*-AEs and [^2^H_4_]-*N*-AEs were synthesized in our laboratory as previously described [35]. Briefly, acyl chlorides were mixed in dry CHCl_3_ with ethanolamine or [^2^H_4_]-ethanolamine, and vigorously mixed at room temperature for 30 min. The resulting solutions were extracted three times with water (GC-MS grade) and the organic layers dried under nitrogen to afford the *N*-AEs and [^2^H_4_]-*N*-AEs, all of which were subsequently purified by solid-phase extraction. The nature and purity of the *N*-AEs and [^2^H_4_]-*N*-AEs were confirmed by TLC and GC-MS.

### Yeast Culture and Homogenate Preparation

Wild-type *S. cerevisiae* cells and mutated yeast strains MATa (BY4741) were maintained on YPD Petri dishes. Some wild-type yeast were transformed with pYES2-Htt25Q-GFP5, pYES2-Htt103Q-GFP5 or pYES2-α-synuclein-GFP5 using the lithium acetate method and were maintained on plates containing synthetic complete media lacking uracil (SC-Ura). Similar transformations were performed in *spo14Δ* and *yju3Δ* yeast strains. Htt103Q is a galactose (GAL)-inducible, FLAG- and GFP-tagged construct encoding the first 17 amino acids of Htt fused to a polyQ tract of 103 glutamines, longer than the threshold for Huntington disease pathogenesis. The *spo14Δ* and *yju3Δ* yeast strains were also transformed with plasmids that express Spo14 and Yju3 (or empty vector), respectively, for reintroduction of the gene of interest. All these expressing strains were grown in SC-Ura media containing raffinose, instead of glucose, and that media replaced by galactose containing media for an overnight induction. Respective yeast strains grown at saturation were recovered by centrifugation (1300×*g*, 10 min, 4°C) and rinsed twice with PBS. Aliquots were then added to glass vials containing glass beads (Sigma, St Louis, MO) and vigorously shaken for 15 min (4°C), allowing for through cell disruption, before determining protein content.

### HEK Cell Culture and Homogenate Preparation

HEK cells were expanded in MEM supplemented with 1 mM glutamine, 10 mM HEPES, 10 mM NaHCO_3_, 100 U/mL penicillin, 100 µg/mL streptomycin and 10% FBS. Confluent cells in 100 mm dishes were rinsed once with PBS, lysed in 1 ml of ice-cold Hepes (250 mM) – Sucrose (10 mM) buffer (pH 7.4) and homogenized on ice using a Dounce tissue homogenizer.

### eCBs and *N*-AEs Quantification by GC/MS

eCBs and *N*-AEs amounts in yeast were quantified as previously described [35]. Briefly, freshly obtained yeast homogenates (3 mg of protein in PBS buffer, except for Figure 1) were added to 10 ml of ice-cold CHCl_3_ containing deuterated standards (200 pmol of [^2^H_4_]-anandamide, [^2^H_4_]-DEA, [^2^H_4_]-HEA, [^2^H_4_]-OEA, [^2^H_4_]-PEA, [^2^H_4_]-SEA and [^2^H_5_]-2-AG). Folch extraction was performed by adding ice-cold CH_3_OH (5 ml) and PBS (total volume of 5 ml). The mixture was vigorously shaken and sonicated (5 min at 4°C) for thorough lipid extraction. Following centrifugation (5 min at 800×*g*), the organic phase was recovered into a glass vial and dried under nitrogen. The residue was dissolved in CHCl_3_ (2 ml) and partially purified by solid-phase extraction using silica and ethyl acetate-acetone (1∶1) as eluent. Eluates were then dried under nitrogen, derivatized using BSTFA (50 min, 55°C), and stored in hexane at −20°C until CI-GC/MS analysis. Calibration curves for eCBs and *N*-AEs quantification were built following the same procedure (except for the absence of yeast homogenate).

### [^3^H]-PEA Hydrolysis

Yeast homogenates (60 µg of protein in 400 µl) or HEK cells homogenate (20 µg of protein in 400 µl) were added to silanized glass tubes containing either 0.5 µl of drug in DMSO or DMSO alone (0.1%, control). Hydrolysis was initiated by the addition of [^3^H]-PEA (100 µl, 70,000 dpm) in Tris.HCl containing 0.1% fatty acid-free BSA. All additions were done using silanized pipette tips. Tubes were incubated for 20 min in a shaking water bath at 37°C. Tubes containing buffer only were used as control for chemical hydrolysis (blank), the value of which was systematically subtracted. Reactions were stopped by the addition of 2 ml of ice-cold CH_3_OH∶CHCl_3_ (1∶1) and the hydrophilic products of the hydrolysis extracted by vigorous mixing and subsequent centrifugation at 800×*g* (10 min). One ml of the upper layer was recovered, mixed with Ecoscint (4 ml) and the radioactivity therein determined by liquid scintillation. Under these conditions, using yeast homogenates, control and blank values were 1078±32 and 410±19 dpm, respectively (values from four experiments, mean±SEM).

### Data Analysis

GraphPad PRISM (version 4) was used to analyze the data and generate dose-response curves.

## Supporting Information

Figure S1 Genetic Modification of PEA Production and Inactivation in Yeast
(0.01 MB PDF)
