# Supplementary material for: Genetic Manipulation of Palmitoylethanolamide Production and Inactivation in Saccharomyces cerevisiae
Source: PLoS One. 2009 Jun 16;4(6):e5942. doi: 10.1371/journal.pone.0005942 (PMC2691958; doi:10.1371/journal.pone.0005942)
Supplement: Figure S1 — Genetic Modification of PEA Production and Inactivation in Yeast (0.01 MB PDF) [file pone.0005942.s001.pdf]

Supplemental Figure 1

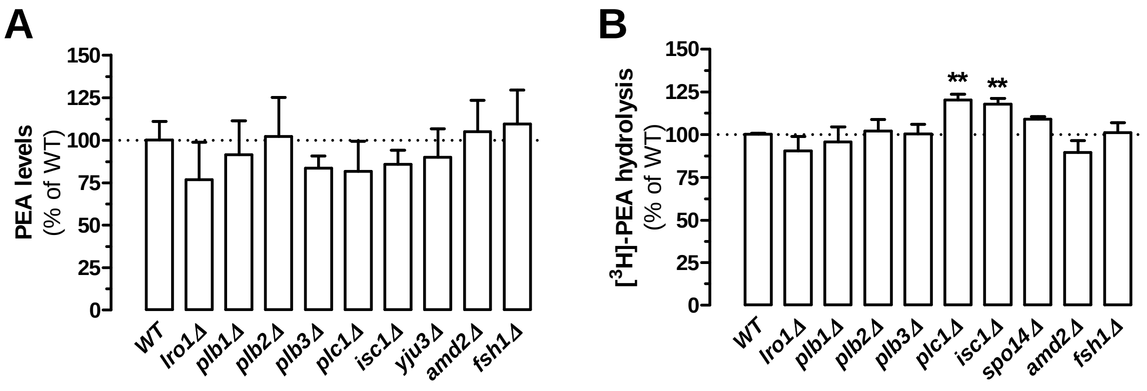

Genetic Modification of PEA Production and Inactivation in Yeast

PEA levels were measured by CI-GC-MS (A) and [^{3}H]-PEA hydrolysis (B) in yeast gene deletion strains compared to those found in a wild-type strain (WT). The following gene-deleted strains were assayed: Δ*iro1*, Δ*plb1*, Δ*plb2*, Δ*plb3*, Δ*plc1*, Δ*isc1*, Δ*spo14*, Δ*yju3*, Δ*amd2* and Δ*fsh1* (Table 1). Values are the mean ± SEM of four to five experiments performed in duplicate (A) and of 3 experiments in duplicate (B), and are expressed as percentage of wild-type yeast. * P<0.05 and ** P<0.01 compared to wild-type yeast (ANOVA one-way, Dunnett’s post test).
